# Pediatric Peritoneal Epithelial Malignant Mesothelioma Case Report

**DOI:** 10.1155/2021/5581757

**Published:** 2021-11-09

**Authors:** Elizabeth Bellew, Samantha Lee, Hiren Patel, Carolyn Fein Levy, Rachelle Goldfisher, John Amodio

**Affiliations:** ^1^Northwell Health-North Shore University Hospital/Long Island Jewish Medical Center, Department of Radiology, New Hyde Park, NY, USA; ^2^Northwell Health-Cohen Children's Medical Center, Department of Pediatric Hematology Oncology, New Hyde Park, NY, USA; ^3^Northwell Health-Cohen Children's Medical Center, Department of Pediatric Radiology, New Hyde Park, NY, USA

## Abstract

We present a 14-year-old boy with peritoneal epithelial malignant mesothelioma (PEMM). While pathology is required to make this diagnosis, radiology plays a crucial role throughout the clinical course of this disease. The key imaging characteristics of peritoneal mesothelioma have been previously well-described in the adult population, but there are rare reports in the pediatric population. This pediatric report highlights the multidimensional use of imaging in this disease, from the initial evaluation to therapeutic supplementation and subsequent follow-up.

## 1. Introduction

Mesothelioma is an exceedingly rare tumor involving the serosal cells of body cavities, occurring 0.5–1.0 cases per 10 million in children. Peritoneal mesothelioma specifically involves the serosa of the peritoneum and represents 20% of these cases. Peritoneal mesothelioma is divided into various subtypes based on clinicopathologic characteristics and consists of malignant mesothelioma, cystic mesothelioma, and well-differentiated papillary mesothelioma. Within the pediatric population, peritoneal epithelial malignant mesothelioma (PEMM) occurs more commonly in females and, unlike the adult population, there is no clear association to asbestos or radiation [1, 2]. Ultrasonography (US) is useful in the evaluation and therapeutic drainage of ascites.

The diagnosis of PEMM is made through pathology and can be supported by molecular testing; however, radiology plays a crucial role throughout a patient's clinical course. In particular, the pretherapeutic assessment of lesions with imaging is crucial to evaluate for resectability. Magnetic resonance (MR) imaging is the imaging modality of choice, as it is efficient in evaluating the extent of disease. Computed tomography (CT) can also be used in the evaluation of these patients who commonly present with abdominal pain. Key imaging findings include peritoneal thickening, nodularity, and enhancement as well as ascites [3]. Positron emission tomography-CT (PET-CT) can help to monitor a patient's response to therapy, which typically consists of a combination of chemotherapy and surgery [4]. This report illustrates the role of imaging throughout the course of PEMM. There is insufficient data on the optimal treatment strategy for managing pediatric peritoneal mesothelioma. The use of combination of neoadjuvant chemotherapy, cytoreductive surgery (CRS), and HIPEC has been reported with favorable outcomes [2].

## 2. Case Report

**Figure 1:**
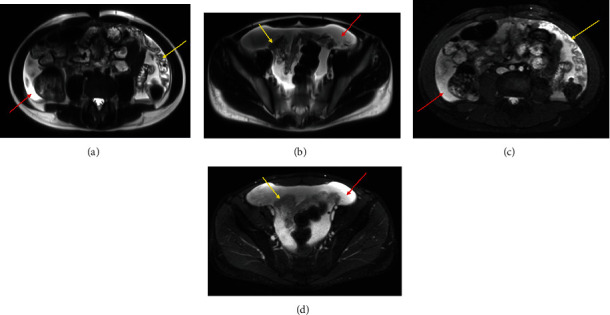
(a, b) Axial T2 images of the midabdomen and pelvis showing ascites (red arrows) and peritoneal thickening (yellow arrows). (c, d) Axial T2 fat-saturated images of the midabdomen and pelvis showing ascites (red arrows) and peritoneal thickening (yellow arrows).

**Figure 2:**
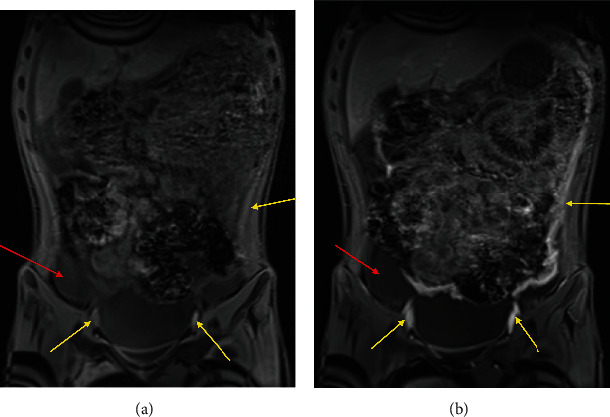
Coronal images before (a) and after (b) the administration of contrast demonstrating enhancement of the peritoneum and omentum.

**Figure 3:**
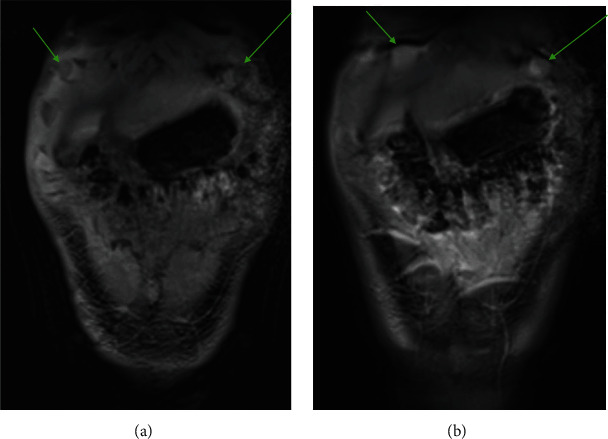
(a) T1 coronal images precontrast and (b) T1 postcontrast images demonstrating enhancing pleural nodules.

**Figure 4:**
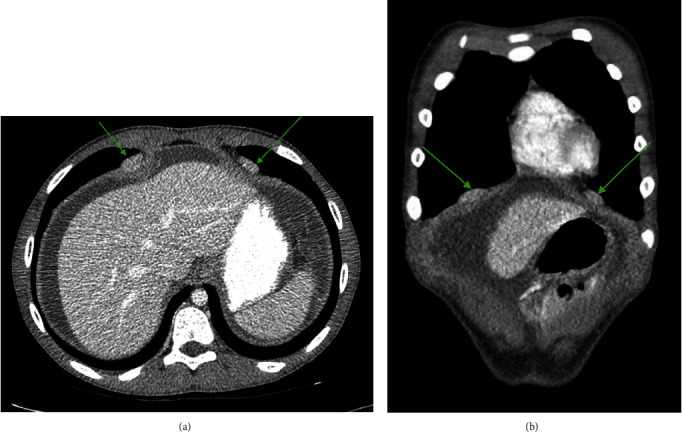
(a) Axial CT with intravenous contrast of the chest demonstrating pleural-based nodules. (b) Coronal CT with intravenous contrast of the chest demonstrating pleural nodules.

**Figure 5:**
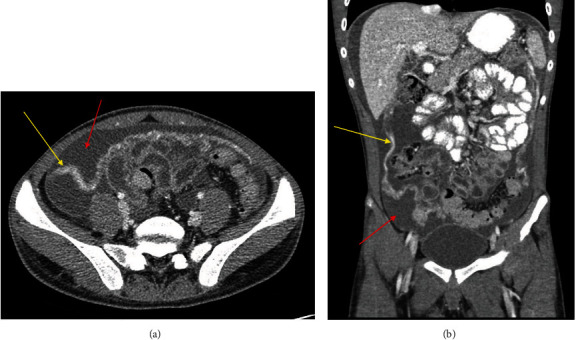
Contrast-enhanced axial (a) and coronal (b) CT images demonstrating omental thickening (yellow arrows) and ascites (red arrows).

**Figure 6:**
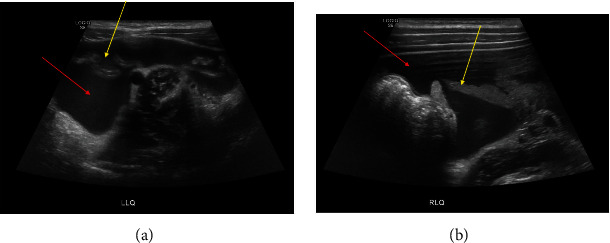
Ultrasound images of the left lower quadrant (a) and right lower quadrant (b) demonstrating ascites (red arrows) and omental thickening (yellow arrows).

A 14-year-old male with a history of growth hormone deficiency, gastroesophageal reflux disease, and asthma presented to his primary care physician with chronic abdominal pain, and over 20-pound weight loss in a 9-month period. C-reactive protein (CRP) and erythrocyte sedimentation rate (ESR) were elevated at diagnosis, 14.6 mg/mL (normal < 4 mg/mL) and 47 (normal < 15), respectively. MR enterography (MRE) of the abdomen and pelvis was performed to evaluate for inflammatory bowel disease, as per our institutional MRE protocol: Breeza was administered orally for small bowel distention and 4 cc of intravenous contrast was administered based on patient weight. MRE revealed a thickened omentum along with diffuse, smooth peritoneal enhancement and thickening. A moderate to large volume of ascites was also present (Figures 1 and 2). No mass was visualized. MRE also revealed enhancing nodules along the superior surface of the diaphragm (Figure 3). The nodules were further evaluated with a dedicated CT chest, abdomen, and pelvis with intravenous contrast that showed two nodular masses along the anterior surface of the diaphragm, suspicious for a mesothelial process (Figure 4) and thickened, enhancing omentum and ascites (Figure 5). Ultrasound of the abdomen redemonstrated a thickened omentum with complex ascites due to tumor (Figure 6).

After initial imaging, interventional radiology performed an ultrasound-guided omental core biopsy and diagnostic paracentesis. The core biopsies consisted of scant fibroconnective tissue. The cytology slides and cell block revealed a hypercellular specimen composed of benign and reactive mesothelial cells, scattered macrophages, and moderate inflammation, but the sample was deemed too small to be diagnostic. Oncology was consulted, and tumor markers were done. CA-125 was elevated at 171 U/mL (normal < 38 U/mL), while CA 19-9, CEA, Beta-HCG, and alpha-fetoprotein were normal.

**Figure 7:**
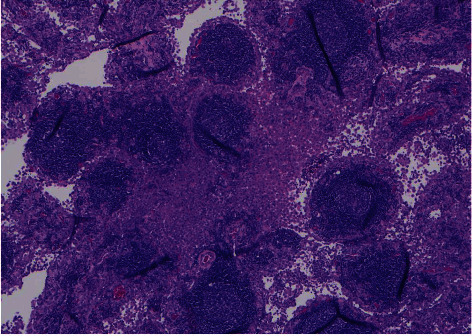
Sheets of neoplastic mesothelial cells with foci of chronic inflammation (pictured). Stains for calretinin, CK 5/6, and WT-1 were positive, confirming mesothelioma (not shown in this image).

Subsequently, laparoscopic omental and peritoneal biopsies were performed to evaluate for inflammatory diseases, malignancy, and possible infection. The biopsy specimen revealed sheets of neoplastic mesothelial cells with foci of chronic inflammation (Figure 7). Stains for calretinin, CK 5/6, and WT-1 were positive, confirming a diagnosis of mesothelioma. Stains for claudin-4, a very broad spectrum carcinoma marker that does not cross-react with mesothelial cells, were negative. Molecular testing of the tumor confirmed CDKN2A homozygous and heterozygous deletion.

**Figure 8:**
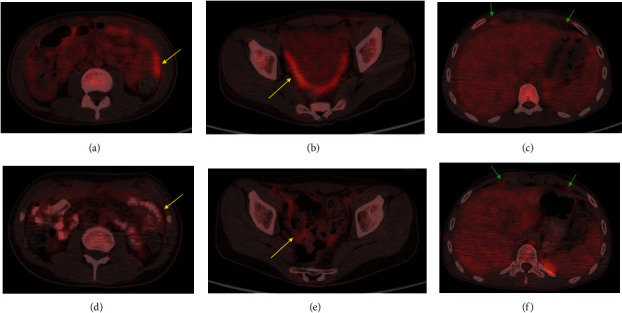
Top images (a–c): initial PET/CT demonstrating FDG avid thickening of the peritoneum and omentum (yellow arrows) and FDG avid nodules along the anterior diaphragm (green arrows). Bottom images (d–f): PET/CT 2 months later with decreased FDG avidity of the peritoneum and omentum (yellow arrows) and nodules along the anterior diaphragm (green arrows).

**Figure 9:**
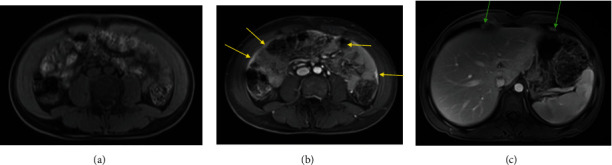
MRI of the abdomen and pelvis three months after the patient's initial MR left T1 precontrast (a), middle T1 postcontrast (b), and right T1 postcontrast (c) showed persistent but decreased peritoneal thickening/enhancement. Pulmonary nodules are decreased in size. No significant ascites is seen.

The patient subsequently underwent 5 cycles of intravenous (IV) chemotherapy with cisplatin, pemetrexed, and Avastin. His tumor was negative for PDL1 and mismatch repair protein defects. Next-generation sequencing (NGS) failed due to inadequate specimen; therefore, we could not assess tumor mutational burden (TMB). Because of a negative PD-L1 status, we did not use PD-L1 inhibitors [5]. After one cycle of IV chemotherapy, the first PET/CT was obtained to evaluate disease burden and for management planning, which revealed FDG avidity of the thickened peritoneum and pleural-based nodules along the diaphragm (Figure 8). Follow-up PET/CT after the third cycle of IV chemotherapy demonstrated decreased FDG avidity in the omentum, peritoneum, and pleural nodules (Figure 8). Follow-up MRI after 5 cycles of IV chemotherapy had persistent but decreased peritoneal thickening/enhancement with resolution of ascites, and the diaphragmatic nodules were decreased in size (Figure 9).

The patient underwent debulking surgery with hyperthermic intraperitoneal chemotherapy (HIPEC) with cisplatin. Due to persistent active disease on pathology in the first debulking surgery, the patient had a second HIPEC with cisplatin with peritoneal catheter placement followed by early postoperative intraperitoneal chemotherapy (EPIC) with 4 cycles of intraperitoneal (IP) cisplatin alternating with mitomycin [1, 6]. Of note, there was no evidence of active disease found during the second HIPEC. Following his 4 cycles of IP chemotherapy, his MRI showed new peritoneal nodularity and large volume ascites concerning for relapsed disease. However, his PET showed no avidity. This discrepancy was resolved by performing a paracentesis which showed no malignant cells, and it was determined the nodularity represented postsurgical and IP chemotherapy-related changes to the peritoneum. In this instance, PET/CT proved to be a useful adjunct to MRI during disease surveillance. The patient's current follow-up is MRI every 4 months to monitor for disease recurrence. To date, there is no evidence of disease after one year of therapy.

## 3. Discussion

Primary peritoneal malignancies, like PEMM, are exceedingly rare but an important diagnosis to consider in cases of pediatric peritoneal pathology. The initial symptoms of mesothelioma can be nonspecific, so diagnosis is often delayed. As in our patient, the initial workup for the nonspecific symptoms including nonspecific elevated inflammatory markers may be targeted more toward looking for inflammatory bowel disease and it can take time to come to the correct diagnosis [3]. Imaging can play a key in the initial consideration of the disease and for monitoring disease response to therapy.

Of note, while imaging clearly plays an important role in the clinical course of PEMM, pathology is required to confirm the diagnosis of peritoneal mesothelioma. Ultrasound-guided biopsies must be supplemented with surgical biopsies in PEMM, as in our case. The molecular testing helped confirm the diagnosis. Studies have shown deletions of the CDK2NA (p16; 9p21) locus in patients with a malignant pleural and peritoneal mesothelioma, whereas deletions of this region were not observed in patients with reactive mesothelial hyperplasia [7]. Germline mutations in BAP1 confer an increased risk to malignant mesotheliomas; however, the clinical course is often less aggressive. BAP1 is also a frequent somatic event observed in patients with pediatric peritoneal mesothelioma [8–10]. Our patient did not have any germline mutations including BAP1 mutation and his NGS failed, so it is unclear if his tumor had a somatic BAP1 mutation, but less likely given normal germline testing.

There are a number of key imaging features of PEMM, which can be seen across various imaging modalities. CT, MR imaging, and US reveal thickened peritoneum; peritoneal nodules; omental, mesenteric, and serosal surface plaque-like masses; and ascites [11]. However, MR imaging, with an emphasis on contrast-enhanced, fat-saturated sequences, is the modality of choice. Fat saturation highlights the extent of peritoneal involvement by suppressing the signal in subcutaneous and intraperitoneal fat, decreasing motion artifact, and removing chemical shift artifact [12]. Fluid-sensitive sequences differentiate ascites from thickened peritoneum [12]. Contrast is also essential for differentiating peritoneal involvement from nonenhancing ascites and surrounding soft tissue; delayed sequences best demonstrate the peritoneal thickening and enhancement [12]. Once a diagnosis of PEMM has been made, hybrid PET/CT can be used as a useful adjunct to assess the primary lesion, detect metastasis, evaluate treatment response, and restage the malignancy following therapy [4]. Pediatric peritoneal mesothelioma has a poor prognosis but may have a better prognosis when compared to adults [13]. Therefore, in addition to chemotherapy, aggressive cytoreductive surgery and intraperitoneal chemotherapy are considered [11].

The limitations of the case include that this is the report of an individual's disease process and treatment, and these things may vary depending on the patient. The value of this report is to make clinicians aware of the entity, although the details within this particular case may not be generalizable to a cohort. While radiology findings can support or suggest the diagnosis, they cannot be used alone and must have pathology and molecular testing for final diagnosis.

Although quite rare, a diagnosis of peritoneal mesothelioma should be considered when presented with the imaging findings illustrated in this case. An interdisciplinary approach is essential for successful diagnosis and treatment of PEMM. The radiologist should be familiar with key imaging findings among various imaging modalities in order to help guide initial investigation and subsequent management of this disease. This report demonstrates the valuable role of the radiologist, the typical radiographic features, and the numerous mechanisms of imaging in a case of PEMM.

## Data Availability

The patient data/case information supporting this case report is not available due to patient privacy. The other information cited in the study is from previously reported studies and datasets, which have been cited in the references.
